# Cellobiose-coated poly (lactide-co-glycolide) particles loaded with diphtheria toxoid for *per os* immunization

**DOI:** 10.3325/cmj.2015.56.85

**Published:** 2015-04

**Authors:** Tetiana Chudina, Andrii Labyntsev, Kyrylo Manoilov, Denys Kolybo, Serhiy Komisarenko

**Affiliations:** 1 Palladin Institute of Biochemistry of the National Academy of Sciences of Ukraine (NASU), Kyiv, Ukraine; 2 Educational and Scientific Center “Institute of Biology,” Taras Shevchenko National University in Kyiv, Kyiv, Ukraine

## Abstract

**Aim:**

To evaluate the dose-dependent immunogenic properties of poly (lactide-co-glycolide) (PLGA) particles coated with cellobiose as antigen carriers for oral immunization.

**Methods:**

Two types of PLGA-cellobiose particles (PLGA-cellobiose-1, ~ 0.8 μm and PLGA-cellobiose-2, ~ 1.2 μm) containing non-toxic recombinant subunit B (SbB) of diphtheria toxin fused with enhanced green fluorescent protein were characterized *in vitro* for their size, shape, antigen loading, and ability to induce phagocytosis. Different doses of antigen, immobilized on the particles (2.5 μg, 25 μg, 250 μg, and 2500 μg per 1 kg of body weight), were administered *per os* 3 times with intervals of 2 weeks to BALB/c female mice. The antigen-specific IgG and IgA antibodies were estimated in serum by ELISA.

**Results:**

After the first immunization, increase in concentration of blood antitoxic antibodies was detected. Antigen dose 250 μg/kg was the most immunogenic for IgG antibodies induction for both types of PLGA-cellobiose particles. Antigen doses 25 μg/kg and 2.5 μg/kg were the most immunogenic for IgA antibodies induction by PLGA-cellobiose 1 and 2 particles, respectively. The second and the third treatment had no significant effect on the immune response or even reduced it, which could be explained by immune tolerance induction by the antigens delivered *per os*.

**Conclusion:**

Our results suggest that the correct dose of PLGA-cellobiose particles loaded with antigen could significantly increase the humoral immune response against the introduced antigen already after the first immunization. Thus, PLGA particles can be considered as a potent component of oral vaccines.

Respiratory diphtheria is a very severe disease caused by toxigenic strains of *Corynebacterium diphtheria* (1). Until the reemergence of diphtheria epidemic in the former Soviet Union in the 1990s, it was considered a well-controlled vaccine-preventable disease. Nowadays, despite the effective childhood immunization program, the majority of the adult population in Europe have no immune protection against this infection (2,3). Therefore, a new effective vaccination strategy against diphtheria is desirable.

Diphtheria toxin (DT) is the main pathogenic factor of *C. diphtheria* (4). This protein consists of two fragments: A (active) and B (binding), which are responsive for toxic effect and cell targeting, respectively. DT can kill mucosal cells at the sites of bacterial colonization and cause systemic reaction in sensitive organisms after penetration into the blood. Specific antibodies can block specific binding of DT to cell receptor and protect the cell and the body from DT toxin action. Therefore, antitoxic antibodies (antitoxins) play the most important role in the immunity against diphtheria. Only antibodies against B-subunit of the toxin have protective properties, because these antibodies can inhibit the toxin binding to the DT receptor.

All current diphtheria vaccines are delivered by parenteral route. In vaccinated persons, they can induce high levels of serum antitoxin, mainly of IgG class, which can prevent systemic spread of the toxin in the case of infection. On the other hand, IgA antibodies play a more important role than IgG antibodies in the protection of mucosal surfaces of the body from mucosal-associated pathogens, like *C. diphtheriae* (5).

Mucosae have their own local immune system known as MALT (mucosa-associated lymphoid tissue), which is able to develop an immune response or tolerance for antigens passing through the mucosal epithelium. Mucosal epithelium contains a specialized cell type, known as M-cells (microfold cells). They can transport different particles like microorganisms and viruses from the mucosal surface to immune cells across the epithelial barrier and thus stimulate mucosal immunity (6). Such function of M-cells could be used in experimental procedures for the delivery of different antigens from mucosa to the immune system. The antigens could be delivered there by oral, nasal, vaginal, or other types of non-invasive vaccination (7).

Oral administration of antigens is considered the most patient-friendly way of immunization (8). However, the efficacy of oral administration of free antigens is limited by their degradation in the gastrointestinal tract and poor adsorption by M-cells. The development of new vaccines against diphtheria depends on the identification of antigens and new routes of immunization (9). It is expected that poly (lactide-co-glycolide) (PLGA) particles would be more appropriate adjuvant for anti-diphtheria vaccines than conventional alum due to their better efficiency, longer potency, and fewer side effects (10). The aim of this study was to assess the ability of cellobiose-coated PLGA particles carrying DT B-subunit to induce local and systemic humoral response after *per os* immunization of mice.

## Materials and methods

### Antigen preparation

Fusion protein EGFP-SbВ consisting of the non-toxic recombinant subunit B of DT (SbB) and enhanced green fluorescent protein (EGFP) was used as a model antigen for immunization of mice. EGFP label was used for further monitoring of its antigen-adsorbing process by different fluorescent techniques.

Recombinant protein EGFP-SbВ was obtained as previously reported (11). Briefly, bacterial culture of *Escherichia coli* BL 21 (DE3) Rosetta (Novagen, Reno, NV USA) transformed by genetic constructions based on plasmid vector pET-24a(+) (Novagen) was grown at 37°C under intensive stirring (250 rpm) up to extinction A_600_ – 0.5-0.7. Expression of the proteins was triggered via incubation with 1 mM of isopropyl β-D-1-thiogalactopyranoside (IPTG) up to 3 hours at 30°С under intensive stirring (250 rpm). Recombinant protein was purified on the Ni^2+^-NTA-agarose column and stored in PBS, рН = 7.2.

### Preparation of PLGA particles

Two types of PLGA particles were prepared and characterized as previously described (12) with slight modifications:

(1) The PLGA particles of the first type (PLGA 1) were prepared by solvent displacement method. Briefly, 3 mL of 0.5% w/v PLGA (lactide:glycolide – 65:35) (Sigma-Aldrich Co., St. Louis, MO, USA) in acetone were added drop-wise to 30 mL of pure distilled water under magnetic stirring. Acetone was evaporated overnight at room temperature. Afterwards particles suspension was filtered through 10 μm filter.

(2) The PLGA particles of the second type (PLGA 2) were prepared by double emulsification using solvent evaporation method. Briefly, 5 mL of 6% w/v PLGA in methylene chloride were homogenized with 1 mL of PBS at 14 000 rpm for 3 min with MPW-309 homogenizer (Universal Laboratory Aid, Warsaw, Poland). Afterwards, the water-in-oil emulsion was added to 150 mL of distilled water containing dioctyl sulfosuccinate surfactant (Sigma-Aldrich Co.) (0.5% w/w PLGA) and homogenized at 7500 rpm for 10 min in an ice bath. Methylene chloride was evaporated overnight at room temperature, after which particles suspension was filtered through 10 μm filter.

### Loading of protein antigens on PLGA particles

PLGA 1 and PLGA 2 particles were collected by centrifugation at 12 000 g for 3 min and resuspended in PBS contained EGFP-SbВ (1% w/w PLGA). The emulsion was stirred overnight on magnetic stirrer at 4°С. Afterwards, the particles were collected by centrifugation for 3 min at 12 000 g. In order to increase the stability, the purified particles were coated in 129 mM cellobiose (Sigma-Aldrich Co.) in PBS for 24 h at 4°C. The suspension of particles was centrifugated for 3 min at 12 000 g, the pellet was resuspended in 1 mL of PBS and stored at 4°С.

### Electrophoretic separation of proteins

10% SDS polyacrylamide gel electrophoresis was performed in compliance with the methodology of Schagger and von Jagow (13).

### Characterization of PLGA particles

Morphological examination of the particles was performed with a transmission electron microscope (TEM) Hitachi H-600 (Hitachi, Japan). The mean size of antigen-loaded PLGA particles was determined using a laser diffraction-based particle size analyzer Zetasizer-3 (Malvern Instruments Co, Malvern, UK). The results were processed using computer software service PCS-Size mode v. 1.61 (Malvern Instruments).

### J774 cell line culture

The murine macrophage-like cell line J774 was obtained from the Cell Cultures Bank of Kavetsky Experimental Pathology Oncology and Radiobiology Institute of NAS of Ukraine. J774 cell line was cultivated in RPMI-1640 medium containing L-glutamine with addition of 5% FCS (fetal calf serum), streptomycin (100 mg/L), penicillin (10 000 U), and amphotericin В (250 μg/L) at 37°С, 5% СО_2_ concentration.

### Flow cytometry

J774 cells were detached from the flask by addition of 20 mM ЕDTA in PBS. Optimal quantity of cells for staining was 0.3-0.5 × 10^6^ per probe. To investigate sorption efficiency of particles, J774 cells were stained by incubation with particles, which contained 3 μg of protein in 1 mL BSA-PBS-NaN_3_ solution (1% BSA and 0.02% NaN_3_ in PBS) for 15 min at 4°С. To investigate phagocytic efficiency, cells were incubated with particles that contained 3 μg of protein in 1 mL BSA-PBS solution (1% BSA in PBS) for 60 min at 37°С. Thereafter, non-bound particles were washed up twice by BSA-PBS solution. Fluorescence intensity of cells was determined by Coulter Epics XL flow cytometer (Beckman Coulter, Brea, CA. USA) via FL1 channel (515-535 nm).

### Preparation of cell specimens for confocal microscopy

J744 cells were grown on microscope coverslips to semiconfluent state and were washed by RPMI-1640, pH 7.3. Particles containing 2.5 μg of protein and cell nuclear dye Hoechst 33342 in 1 mL RPMI-1640 were incubated with cells for 15 min at 4°С and for 60 min at 37°С. Non-bound proteins were washed twice by RPMI-1640. After incubation, J774 cells were fixed by 4% solution of paraformaldehyde in 0.1 M phosphate buffer for 40 min at 4°С. Coverslips with cells were mounted on a slide in mounting medium based on polyvinyl alcohol. Cell specimens were analyzed with confocal microscope Zeiss LSM 510 Meta (Carl Zeiss, Jena, Germany) with oil immersion objective Plan-Apochromat 63x/1.4 Oil DIC.

### Immunization of mice

Immunization was carried out on BALB/c female mice of the same age (6 weeks) and weight ( ~ 20 g) in strict accordance with the recommendations in NIH Guide for the Care and Use of Laboratory Animals. The protocol was approved by the Committee on the Ethics of Animal Experiments of the Palladin Institute of Biochemistry of NAS of Ukraine (Protocol #2 from 19/09/2012).

Animals had free access to food and water during the immunization study. They were deprived of water 24 h prior to immunization. Mice were fed *per os* with 25 µL of soluble PLGA particles functionalized with EGFP-SbВ and cellobiose in PBS. Eight groups of mice (9 mice per group) were immunized orally with different doses of PLGA 1 and PLGA 2 particles having immobilized antigen (2.5 µg, 25 µg, 250 µg, and 2500 µg of antigen per 1 kg of body weight) three times with intervals of two weeks on days 0, 14, and 28. 100 µL of blood samples were taken from the tail vein before immunization and one week after each immunization. Blood samples were centrifuged for 20 min at 2800 rpm. The sera samples were stored at -20°C.

### Determination of antigen-specific IgG and IgA in sera

IgG and IgA antibodies against SbB were determined by enzyme-linked immunosorbent assay (ELISA). Antigen (10 µg/mL in PBS) was adsorbed on the bottom of 96-wells plates (Microlon ELISA-plates, High-Binding, Greiner Bio One, Frickenhausen, Germany) by overnight incubation at 4°C. The plates were washed three times with 0.04% v/v Tween® 20 in PBS (PBST) and blocked with PBS-skim milk powder (1% w/v) for 1 h at 37°C, followed by washing with PBST. The serum samples were serially diluted with PBST, and 100 μL of each sample was added to each well. The plates were incubated for 60 min at 37°C and washed three times with PBST. Afterwards, 100 µL of peroxidase-conjugated anti-mouse IgG/IgA (Sigma) were added according to the manufacturer’s instructions and incubation was repeated. The plates were washed three times with PBST. Then, 100 mL of tetramethyl benzidine solution was added to each well and after 15 min the reaction was stopped by addition of 2N H_2_SO_4_. The optical density (OD) was measured at 450 nm.

## Results

### Preparation and characterization of PLGA particles

**Figure 1:**
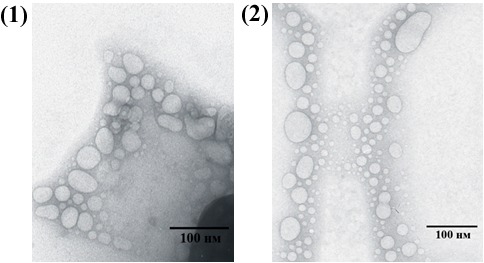
TEM photomicrograph of poly (lactide-co-glycolide) (PLGA) particles samples prepared by methods 1 and 2.

In order to prepare suitable carriers for the delivery of antigen to mice MALT we used two methods of PLGA particles synthesis, which resulted in the formation of particles with different size and structure PLGA 1 were prepared by solvent displacement method and PLGA 2 – by double emulsification with solvent evaporation method. TEM results showed that both types of particles had similar oval to round shape (Figure 1). The final particles mean size and polydispersity index (PDI) after the antigen-loading and cellobiose coating were measured using a Zetasizer. The PDI values for these particles ranged from 0.3 to 0.4, a higher value indicating a less homogeneous particle size distribution. Batches of PLGA-cellobiose-1 had a mean size of 870 nm and a PDI of 0.406, whereas batches of PLGA-cellobiose-2 had a mean size of 1189 nm and a PDI of 0.3. More detailed analysis showed that both types of the particles had heterogeneous distribution of mean size. Thus, the batch of PLGA-cellobiose-1 particles was composed of two populations of particles with distinct mean sizes – 178.6 nm (33%) and 707.1 nm (67%); whereas the batch of PLGA-cellobiose-2 was composed of particles with the mean sizes of 495.2 nm (79%) and 1143 nm (21%). Also, SDS PAGE showed that the amount of antigen entrapped in the PLGA-cellobiose-1 was 15% higher than in the PLGA-cellobiose-2 particles (Table 1). Thus, we obtained particles with immobilized antigen, which considerably increased their size after functionalization with cellobiose.

**Table 1:** Main properties of synthesized poly (lactide-co-glycolide) (PLGA) particles

Properties	PLGA 1	PLGA 2
Size before antigen loading and cellobiose coating, nm	8-35	10-100
Size after antigen loading and cellobiose coating (mean size±SD), nm	870 ± 14	1189 ± 39
Mean size distribution (number %), nm	178.6 (33) 707.1 (67)	495.2 (71) 1143 (29)
Average efficiency of antigen loading (μg/mg)	4.6	9.0
Polydispersity index after antigen loading and cellobiose coating	0.406 ± 0.059	0.300 ± 0.057

### Interaction of PLGA-cellobiose particles with J744 cells

Mouse macrophage cell line J774 was incubated with PLGA-cellobiose particles of types 1 and 2 during 15 min at 4°С to determine the sorption efficiency and during 60 min at 37°С to evaluate the ability of particles to induce phagocytosis by J774 cells. *E coli* cells expressing green fluorescent protein EGFP were used for comparison of phagocytic activity, since they are considered a standard of effective sorption and internalization due to immunotropic properties of lipopolysaccharides and other components of bacterial origin on their surface.

**Figure 2:**
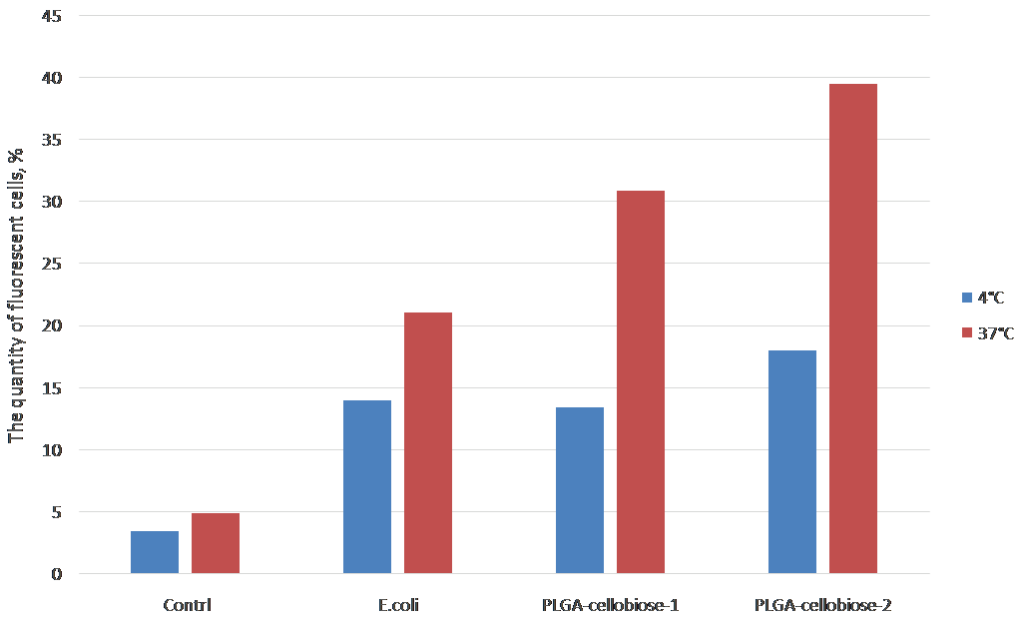
Histograms of phagocytosis efficiency for J774 cells, which were incubated with fluorescent poly (lactide-co-glycolide) (PLGA)-cellobiose particles, *E coli*, or without any fluorescently labeled agent (control) at 4°С (incubation time – 15 min) and 37°С (incubation time – 1h). The amount of positively stained, ie, phagocytic cells was calculated as a gate, which restricted cells with higher than unstained cells fluorescent intensity. Gate was determined with positive control cells, which were incubated with *E coli*.

Cell fluorescence intensity was determined with Coulter Epics XL (Beckman Coulter) flow cytometer. J774 cells effectively adsorbed both types of PLGA-cellobiose particles and bacteria at 4°С and 37°С. Surprisingly, J774 cells interacted with particles better than with bacteria. Also, PLGA-cellobiose-1 particles adsorbed and internalized by macrophage cells on average 10% more effectively than PLGA-cellobiose-2. Thus, J774 cells showed a high uptake efficiency of particles in the range: PLGA-cellobiose-2 – 40%; PLGA-cellobiose-1 – 31%; *E.coli* – 21% (Figure 2). Consequently, we obtained particles highly attractive for immune cells. Moreover, different types of these particles differed in their ability to bind phagocytic cells.

**Figure 3:**
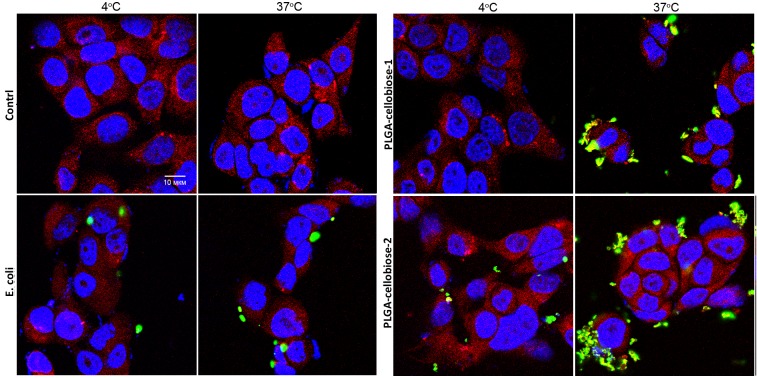
Confocal images of J744 cells incubated with poly (lactide-co-glycolide) (PLGA)-cellobiose particles, *E coli* cells, or without any foreign fluorescently labeled agent (control) at 4°С (incubation time – 15 min) and 37°С (incubation time – 1h). Cell nuclei are stained in blue (Hoechst 33342), cytoplasm is in red (autofluorescence at excitation wavelength of 560 nm) and antigen-loaded PLGA particles and *E coli* cells are stained in green (enhanced green fluorescent protein as label).

J744 cells were fixed after incubation with the particles and were analyzed by Zeiss LSM 510 Meta (Carl Zeiss) confocal microscope (Figure 3). J744 macrophage cells bound PLGA-cellobiose particles on their surface, and the interaction between cells and particles was strong enough to prevent washing them away with buffer solution during the procedure of microscopic slide preparation. The confocal images were in good agreement with the results obtained by flow cytometry.

PLGA-cellobiose particles interacted with J744 cells more effectively than *E coli* cells. Interaction of PLGA-cellobiose-1 particles with J774 cells was weaker at 4°C than at 37°C. In fact, particles of type 1 were almost not bound by J744 cells after incubation at 4°C during 15 min compared to particles type 2, which remained bound by cells under these conditions. Moreover, we showed a more efficient uptake of PLGA-cellobiose-2 particles compared to PLGA-cellobiose-1 by J744 macrophage cells. We believe that this effect appeared due to bigger average size of PLGA-cellobiose-2 particles, which is closer to the natural size of bacteria cells. Based on this, it could be assumed that PLGA-cellobiose-2 particles have better interaction with phagocytic cells *in vivo* and thus possess more intensive immunogenic properties than PLGA-cellobiose-1 particles.

### Immunization of mice and analysis of dose-dependent systemic immune responses

Both types of obtained PLGA-cellobiose particles interacted with macrophage-like cells, as was shown by flow cytometry and confocal microscopy, and did not show any dose-dependent cytotoxic effect *in vitro* (data not shown). By their size and shape these particles resembled bacterial cells. Therefore, we suppose that they should also interact similarly as bacteria with M-cells of Peyer's patches at the small intestine or other possible phagocytic cells of murine MALT. Therefore, these antigen-delivery compositions could possess some defined immunogenic potential and are suitable for studies *in vivo*.

**Figure 4:**
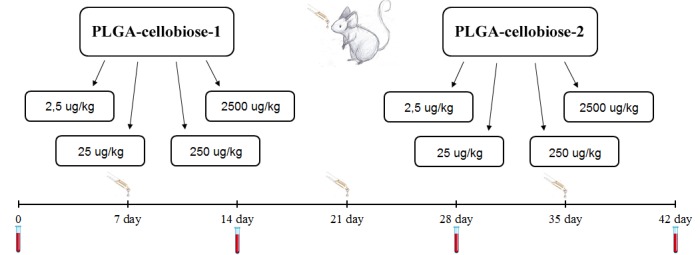
The immunization chart: mice were immunized by two types of antigen- poly (lactide-co-glycolide) (PLGA) -cellobiose compositions: 8 groups (9 mice each) received different doses of PLGA-cellobiose particles type 1 and 2. Blood collection is marked as red tubes and oral immunization is marked as yellow pipettes.

The aim of *in vivo* study was to determine the dynamics of systemic humoral immune response after mice oral vaccination with different doses of antigen-loaded particles. For this purpose PLGA-cellobiose particles 1 and 2 were administered *per os* at days 7, 21, 28 and 35 in doses of 2.5, 25, 250, and 2500 μg of antigen per kilogram of mice body weight. Serum samples were collected at days 0, 14, 28, and 42 after antigen-loaded PLGA particles administration (Figure 4).

**Figure 5:**
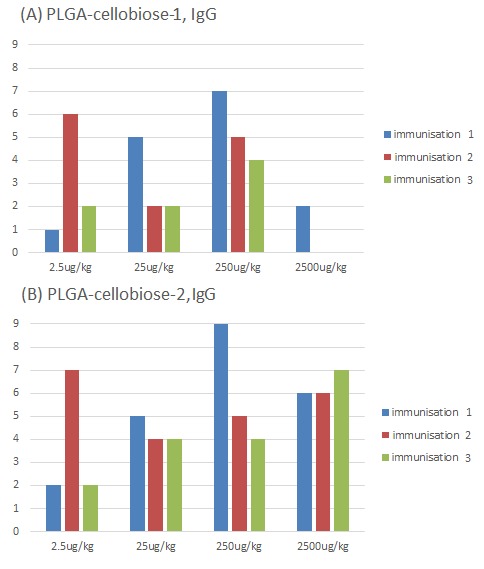
The number of animals with significantly positive levels of serum specific IgG, which were immunized orally with different doses of antigen-loaded to poly (lactide-co-glycolide) (PLGA)-cellobiose-1 (**A**) and PLGA-cellobiose-2 (**B**). The serum was collected at days 0, 14, 28, and 42. The total number of animals per bar was 9.

**Figure 6:**
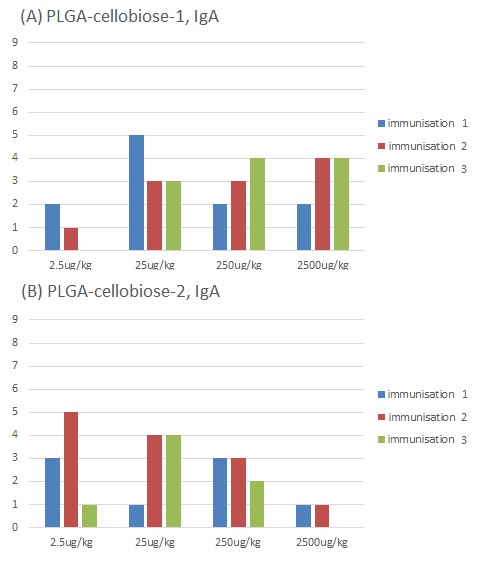
The number of animals with significantly positive levels of serum specific IgA, which were immunized orally with different doses of antigen-loaded poly (lactide-co-glycolide) (PLGA)-cellobiose-1 (**A**) and PLGA-cellobiose-2 (**B**). The serum was collected at days 0, 14, 28, and 42. The total number of animals per bar was 9.

IgG and IgA levels of antibodies in blood of animals were measured by ELISA. The number of animals with considerably increased levels of immunoglobulins was determined for each group (Figure 5 and 6). All groups of animals immunized with PLGA-cellobiose-1 and PLGA-cellobiose-2 particles with the same doses of loaded antigen showed similar pattern of positive responses.

All mice immunized with 250 μg/kg of antigen associated with PLGA-cellobiose-2 particles had significantly elevated levels of antibodies. The titers of antibodies after the first immunization in this group of animals were 1:800 and more in ELISA (data not shown). However, the level of antibodies decreased after the second and third immunizations. The similar tendency was observed for the dose of 250 μg/kg of antigen associated with PLGA-cellobiose-1 particles.

The highest number of animals that had significantly positive levels of specific IgA antibodies was found in two groups: 25 μg/kg PLGA-cellobiose-1 after the first immunization and 2.5 μg/kg PLGA-cellobiose-2 after the second immunization (Figure 6).

Thus, cellobiose-coated PLGA particles of both types were effective for oral immunization. However, after the second administration of PLGA-cellobiose particles a considerable part of immunized mice became tolerant. The number of tolerant animals was increasing after each subsequent administration of particles. This process may represent the immune system tendency to develop an immune tolerance to repeatedly delivered antigens *per os*.

## Discussion

Non-toxic DT mutants (cross-reacting materials) have already been applied in animal models to induce effective anti-diphtheria immune response (14). For example, in our previous study non-toxic recombinant subunit B of DT has been proposed as a safe and effective antigen with a good potential to elicit protective immune response in immunized guinea pigs (15). However, the efficacy of the oral administration of free antigens is limited by their degradation in the gastrointestinal tract and poor absorption by M-cells. On the other hand, pulmonary immunization route could be complicated by the development of the inflammation observed in the lung tissues of the animals receiving antigen (16). Today, there is a number of biocompatible particles with controlled volume that were developed for antigen delivery in *per os* immunization. Biodegradable polymers, like PLGA, are widely used for the design of these delivery vehicles (17). However, precise schemes of immunization and antigen formulations are still to be developed for induction of anti-diphtheria immunity.

In this study, we examined humoral immune responses in mice immunized with DT B-subunit loaded PLGA-cellobiose microparticles. Two types of antigen-loaded PLGA-cellobiose particles were obtained by different methods of synthesis. The particles had different average size and polydispersity index. They also differed by their capability of engulfment of protein antigens and antigen release during their presence in MALT. Therefore, we could expect different immunogenic properties from these two kinds of antigen-delivery systems.

Immune response to the antigen is usually induced by its interaction with antigen-presenting cells (APC) of the immune system, macrophages, and dendritic cells (18).

The monocytic macrophage-like cell line J744 derived from murine reticulum cell sarcoma was used to study particles’ interaction with APC. It was found that sorption of *E coli* cells to J744 macrophages at 4°C for 15 min is almost the same as sorption of PLGA-cellobiose-2 particles and even better than that of PLGA-cellobiose-1. However, internalization of particles of both types was more effective than internalization of *E coli* cells (37°C, 60 min). Thus, effective interaction between obtained antigen-PLGA-cellobiose compositions and APC was demonstrated *in vitro*.

Since synthesized particles could bind to the macrophage antigen-presenting cells *in vitro*, it could be assumed that similar effects would also be observed *in vivo*. Particularly, when particles are administered into the body by immunization, they might interact with APCs of the immune system and stimulate the development of a specific immune response to the antigen (19). Nevertheless, it is also necessary to take into account the ability of the gastrointestinal tract to induce immune tolerance (20). Therefore, we decided to investigate whether our composites maintained their properties and caused immune response when administered orally to mice. Experimental animals were immunized with different types of particles at various doses, which allowed us to find out how the amount of antigen in the particles could influence the development of immune response.

The increase in specific antibodies in blood of animals was observed for both types of particles at any doses examined. Higher level of IgG specific to recombinant subunit B was found when the dose of PLGA-cellobiose-2 particles was administered at 250 μg/kg. All mice in this group had a significant increase in specific IgG level in plasma. Since IgG are the main component of humoral immunity, synthesized PLGA composites could be considered as the effective carrier for the antigen delivery and for induction of humoral immune response. The highest levels of specific IgA were found in the group of animals immunized with 250 μg/kg PLGA-cellobiose-1 particles. Notably, the secretory IgA are the main protective antibodies on mucosal surfaces. The rate of secretory IgA correlates positively with their level in blood. So, the increased levels of IgA can indicate a possible increase in mucosal protection under oral administration.

However, in many cases, upon repeated administration of particles, the level of antigen-specific antibodies was reduced, pointing at the development of oral tolerance. Nevertheless, for the formulation of convenient oral vaccine this tendency is not significant as in this case only one administration of vaccine is required for the development of high immune response and further immunizations are not necessary.

In conclusion, two methods of antigen-PLGA-cellobiose composition were developed and appropriate particles were synthesized according to these methods. The interaction with monocyte-macrophagal antigen-presenting cells *in vitro* and their influence on IgG and IgA response *in vivo* were studied in a mouse model. We found that PLGA conjugated DT B-subunit could induce enhanced levels of antigen-specific IgG and IgA in the serum after first immunization. The mechanisms of particle penetration into the immune cells and their interaction with immune system require further study.
